# Role of Solid Fat Content in Oxidative Stability of Low-Moisture Cracker Systems

**DOI:** 10.3390/antiox11112139

**Published:** 2022-10-28

**Authors:** Thanh Phuong Vu, Cansu Ekin Gumus-Bonacina, Maria G. Corradini, Lili He, David Julian McClements, Eric A. Decker

**Affiliations:** 1 Department of Food Science, College of Natural Sciences, University of Massachusetts Amherst, 102 Holdsworth Way, Amherst, MA 01003, USA; 2 Department of Food Engineering, Faculty of Engineering, Ankara University, 06830 Ankara, Turkey; 3 Food Science Department, University of Guelph, 50 Stone Road East, Guelph, ON N1G 2W1, Canada

**Keywords:** lipid oxidation, interesterified fats, low moisture food, confocal microscopy, antioxidants, fat mobility, luminescent molecular rotors

## Abstract

Lipid oxidation is a major pathway for the chemical deterioration of low-moisture foods. Little is known about how the physical properties of the fat used in crackers impact lipid oxidation kinetics. Fully hydrogenated soybean fat + interesterified soybean oil, fully hydrogenated soybean fat + sunflower oil, fully hydrogenated soybean oil, and soybean oil and interesterified fat alone were formulated to have varying solid fat content (SFC) at 55 °C but the same linoleic acid and tocopherol contents, so the fats had similar susceptibility to oxidation. A fluorescence probe showed that lipid mobility increased with decreasing SFC in both cracker doughs and fat blends, suggesting the probe could be used to monitor SFC directly in foods. Decreasing SFC decreased oxidation in crackers. Crackers made from interesterified fat (13.7% SFC) were more oxidatively stable (hexanal lag phase = 33 days) than crackers made from fat blends (hexanal lag phase = 24 days). These results suggest that blended fats result in regions of liquid oil high in unsaturated fatty acids within a food product prone to oxidation. Conversely, interesterified fats where unsaturated and saturated fatty acids are more evenly distributed on the triacylglycerols are more stable. Thus, interesterified fats could allow for the formulation of products higher in unsaturated fatty acids to improve nutritional profiles without sacrificing shelf life.

## 1. Introduction

Fats and oils play an important role in the structure, mouthfeel, and flavor of bakery products [1]. Fats and oils are susceptible to lipid oxidation, which often determines the shelf life of these food products. Lipid oxidation starts when a hydrogen is removed from an unsaturated fatty acid to form an alkyl radical that then reacts with oxygen and other fatty acids to form lipid hydroperoxides. These hydroperoxides then decompose, resulting in fatty acid scission to form compounds responsible for off-flavors known as rancidity. Many factors influence the rate of lipid oxidation, including degree of fatty acid unsaturation, type and concentration of prooxidants and antioxidants, and the physical state of the food system [2].

Blends of different fats can result in changes in oxidative stability compared to each of the individual fats alone. The hydroperoxide lag phase of sunflower oil and rice bran oil increased after coconut oil was added to these two oils [3]. A blend of soybean oil and high-oleic sunflower oil or a blend of canola oil and high-oleic sunflower oil had higher oxidative stability than partially hydrogenated soybean oil or partially hydrogenated canola oil with the same amount of linolenic acid [4]. In addition, the oxidative stability of fish oils could be increased by blending them with wheat germ oil, medium-chain triglycerides, and high-oleic sunflower oil [5,6]. Blending can increase the oxidative stability of oils high in polyunsaturated fatty acids because polyunsaturated fatty acids become diluted, i.e., their overall concentration is lower in a studied system, when blended with a saturated or monounsaturated fat source. In addition, oxidative stability can increase when one of the fats has a high antioxidant concentration, which can protect a second fat that is low in antioxidants. 

Fat blends, however, can have limited applications in some foods since they do not provide desirable physical characteristics due to uneven melting profiles and separation of solid and liquid phases. Fats produced by interesterification are designed to improve the physical properties of blended fats because the rearrangement of fatty acids in the triacylglycerols results in more even melting behavior. While interesterified lipids have desirable physical characteristics, in many cases, their oxidative stability has been reported to decrease [7]. The interesterification and post-process purification methods are important factors because these operations can cause oxidation and/or decrease antioxidant concentrations. Fat source, degree of unsaturation, and position of fatty acids on the glycerol backbone can also influence the oxidative stability of the interesterified lipids [8,9]. 

Both blending and interesterification yield physical changes that can impact lipid oxidation, yet there have been few studies regarding the influence of oxidative stability of fat blends and interesterified fats once they are incorporated into food products. The exception is research in oil-in-water emulsions that show that the presence of solid fat could increase oxidation if the fat crystalizes and drives the migration of liquid fat onto the surface of the oil droplet, where it is easily oxidized [10,11,12]. Alternately, research has shown that when a solid fat layer forms a shell at the emulsion droplet interface (e.g., crystallization of surfactants), it can inhibit lipid oxidation by decreasing the ability of water-soluble prooxidants to interact with unsaturated lipids in the core of the emulsion droplet [13,14,15,16]. 

Physical structure of low-moisture food systems is dramatically different than either bulk oils or oil-in-water emulsions, and this may lead to different oxidation mechanisms. Labuza (1970) described low-moisture foods as those that have a water activity less than 0.5 [17]. The rate of lipid oxidation in low-moisture model crackers is affected by environmental parameters differently than in foods such as emulsions, bulk oils, or meats. For example, the lag phase of hexanal formation is much longer than the lag phase of hydroperoxides formation compared to emulsions, bulk oils, or meats, where these two oxidation products have similar lag phases [18]. This suggests that the physical structure of crackers makes hydroperoxide more stable because the low diffusion conditions make it difficult for iron promote the decomposition of the hydroperoxides. Furthermore, iron shows little impact on the oxidative stability of crackers, but it is a strong pro-oxidant in bulk oils and oil-in-water emulsions [18]. Oxidation rates decrease with decreasing water activity [19]. Hydrophobicity of antioxidants also showed different trends in effectiveness in crackers, where very hydrophobic 20-carbon antioxidant esters were most effective [18] compared to both emulsions, where surface-active antioxidants (8- and 12-carbon antioxidant esters) are most effective [20], and bulk oils, where hydrophilic antioxidants are most effective [21]. Reducing sugars were found to inhibit lipid oxidation in crackers better than proteins [19]. Surface lipids were not found to oxidize faster than total lipids in crackers, which is different than spray-dried emulsions, where surface lipids oxidize first [22]. Moreover, it was shown that the initial oxidative state of fats used as ingredients for cracker production significantly affects the rancidity and shelf life of the final product [23].

As with oil-in-water emulsions, the oxidative stability of low-moisture foods may depend on the physical arrangement of fat. For example, solid and liquid fat can form multiple phases with different levels of oxidative stability due to different degrees of unsaturation. Additionally, the fat phase might be able to interact more or less with antioxidants present in the food depending on how the components are physically arranged within the food. Confocal microscopy shows that in low-moisture cracker systems, fat formed a continuous phase surrounding starch [18]. It is unknown if altering solid fat content could influence the organization of lipids in relation to other components in cracker systems, which in turn could impact their oxidative stability.

To our knowledge, no systematic research has been conducted on how solid fat content impacts the oxidative stability of low-moisture foods. This is important since the type of fat used in these products has recently changed due to the removal of partially hydrogenated fats, which are no longer listed as generally recognized as safe (GRAS) food ingredients by the United States Food and Drug Administration [24]. Products such as crackers need solid fat to develop a desirable and stable physical structure and decrease the migration of the fat out of the product. Altering the ratio of saturated and unsaturated fatty acids in triacylglycerols by interesterification (IE) can result in fats with the high solid fat content (SFC) needed for baked goods while also having high amounts of unsaturated fatty acids that are beneficial for health. Thus, with the removal of partially hydrogenated fats from the food supply, interesterified fats are often the preferred form of fats for low-moisture foods such as crackers.

In this study, we aim to determine how SFC affects lipid oxidation in crackers. We hypothesize that fractions of solid and liquid fat could alter physical properties, thus potentially changing oxidative susceptibility. Experiments were performed using fat blends that varied in SFC yet had similar polyunsaturated fatty acid and tocopherol content. To better understand how SFC impacts fat distribution, a lipophilic fluorescent molecular rotor, 1-(2,5-dimethoxy-phenylazo)-naphthalen-2-ol (citrus red 2), was used to characterize fat mobility in the cracker dough, and fat-distribution images were captured using confocal microscopy.

## 2. Materials and Methods

### 2.1. Materials

Fully hydrogenated soybean oil, interesterified soybean oil, and regular soybean oil were provided by Archer Daniels Midland Company (Decatur, IL, USA). Sunflower oil was purchased from a local grocery store.

Sodium hydroxide, sodium sulfate anhydrous, hydrochloric acid, methanol, hexane, isopropanol, and chloroform were purchased from Fisher Scientific (Hampton, NH, USA). Dioxane was purchased from Acros Organics (Fair Lawn, NJ, USA). Boron trifluoride in methanol (14% *w*/*w*), ferrous sulfate, barium chloride, ammonium thiocyanate, and cumene hydroperoxide; α-, ɣ-, and β- tocopherols; and 1-(2,5-dimethoxy-phenylazo)-naphthalen-2-ol (citrus red 2) and hexanal were purchased from Sigma-Aldrich (St. Louis, MO, USA). BODIPY 493/503 was purchased from Invitrogen (Waltham, MA, USA). 

### 2.2. Methods

#### 2.2.1. Fatty Acid Analysis

Fats (20 mg) were saponified with 0.4 mL of 0.5 N NaOH in methanol at 100 °C for 5 min in 15 mL screw-cap glass tubes [25]. Samples were cooled down to room temperature, and 0.4 mL boron trifluoride in methanol (14%) was added; then, the samples were heated again to 100 °C for 5 min to methylate the fatty acids. Following the methylation, samples were cooled down, and 1 mL hexane was added, followed by the addition of 8.5 mL double-distilled water. Glass tubes were placed in a benchtop rocking shaker for 10 min, and the mixtures were centrifuged at 1000× *g* for 5 min. The organic layer was transferred to a 1.5 mL vial containing an approximately 1 mm layer of anhydrous sodium sulfate. Vials were flushed with nitrogen and kept at −20 °C until fatty acid analysis was conducted.

Fatty acid profile was assessed using a QP2010 SE Shimadzu GC/FID/MS (Tokyo, Japan). The chromatographic separation of fatty acid methyl esters was performed on a Supelcowax 10 column (100 m × 0.25 mm i.d. × 0.25 μm). The initial oven temperature was 50 °C. The oven temperature was then increased 20 °C/min to 200 °C, increased 2 °C/min to 220 °C, and finally held at 220 °C for 142.5 min. The temperature of both the injector and flame ionization detector was 250 °C. Air, helium, and hydrogen pressures were 80, 120, and 80 psi, respectively. Purge flow was 3 mL/min, and column flow was 1.5 mL/min. Samples (1 μL) were injected at a split ratio of 10:1. Fatty acid methyl esters were identified by comparing retention times with standards or by their mass spectra and further compared with the National Institute of Standards and Technology Mass Spectra Library. Based on identified peaks, percentages of fatty acids were calculated by GC/MS post-run analysis software [26].

#### 2.2.2. Measurement of Tocopherol Homologs 

Fat samples (50–60 mg) were dissolved in 10 mL hexane and filtered with a 0.2 μm syringe filter (Fisher Scientific, Pittsburgh, PA, USA). Each sample (20 μL) was then injected into a Shimadzu HPLC system equipped with a Supelcosil LC-Diol column (250 mm × 4.0 mm, 5 μm). The mobile phase was a mixture of hexane and dioxane (19:1 *v*/*v*), and the flow rate was 1 mL/min. A Shimadzu RF-20Axs fluorescence detector was used to detect tocopherol homologs at an excitation wavelength of 290 nm and an emission wavelength of 330 nm. Peak integration was performed using Shimadzu EZstart software (version 7.2). Tocopherol homologs in the samples were identified and their concentration calculated based on standard curves prepared with the individual tocopherol homologs.

#### 2.2.3. Solid Fat Content Measurement

The solid fat content (SFC) was measured using a Q100 TA Instruments differential scanning calorimeter (New Castle, DE, USA), based on a method described by Márquez, Pérez, and Wagner [27]. Fats and fat blends (5–10 mg) were loaded into aluminum pans and sealed with aluminum lids (TA Instruments, New Castle, DE, USA). An empty pan and lid were used as a reference for each measurement. Samples were cooled to −90 °C at 10 °C/min, held at −90 °C for 10 min, and heated up to 80 °C at 5 °C/min. Samples were measured in duplicate. From the thermograms, the absolute melting energy (E) was measured, and the SFC at a selected temperature (T) was calculated as indicated below (Equation (1)), where dT represents the change in temperature:

(1) $${\mathrm{SFC}}_{T}=\frac{\int_{T}^{80}\mathrm{EdT}}{\int_{-90}^{80}\mathrm{EdT}}\times 100$$

#### 2.2.4. Fat Blends

Based on the fatty acid profiles of fully hydrogenated soybean oil, interesterified soybean oil, regular soybean oil, and sunflower oil, fat blends were formulated to have equal linoleic acid (18:2) concentrations. In addition, tocopherol concentrations in the blended fats were determined and individual tocopherol homologs were added to make the total antioxidant concentration and tocopherol homolog composition equal in all samples. 

All blends were prepared by first melting individual fats completely at 90 °C, then adding fats together and stirring for 5 min. Individual tocopherol homologs were added after cooling. The final fat blends were stored at −20 °C until use.

#### 2.2.5. Cracker Preparation and Storage

Cracker preparation was adapted from Barden et al. [18]. A mixture of flour (50.6 wt.%), salt (1.2 wt.%), and baking soda (0.5 wt.%) was added to the fat (8.1 wt.%). Ingredients were mixed by a Kitchen Aid mixer (model #KSM95, Mississauga, ON, USA) for 2 min. Water (31.5 wt.%) was added, and the resulting dough was kneaded by hand with the incorporation of additional flour (8.1 wt.%) to minimize sticking. The dough was flattened by a pasta roller (Kitchen Aid KPSA attachment, Mississauga, ON; thickness setting 2, approximately 3 mm) and cut into 2.5 cm × 2.5 cm pieces before baking at 163 °C for 21 min. After baking, crackers were crumbled using a mortar and pestle; distributed (0.5 g) into acid-washed, 10 mL glass GC vials (Supelco Analytical; Bellefonte, PA, USA); and stored at 55 °C in the dark.

#### 2.2.6. Measurement of Lipid Oxidation

Lipid hydroperoxide content was measured using a colorimetric ferric thiocyanate method [28]. Ferrous sulfate (0.1 g) was dissolved in 10 mL double-distilled water. Barium chloride (0.2 g) was dissolved in 25 mL of 0.4 N HCl. The ferrous sulfate solution was mixed with barium chloride solution at a ratio of 1:1 *v*/*v* and centrifuged at 3400× *g* for 3 min, with the supernatant being used as described below. This solution was prepared fresh each day.

Cracker samples (0.100 ± 0.005 g) were weighed into 10 mL disposable test tubes. A mixture of chloroform and methanol (2:1 *v*/*v*; 0.5 mL) was added to each sample and centrifuged (3400× *g*) for 10 min, and 200 µL of the supernatant was mixed with 16.7 μL of the ferrous solution, 3.2 mL of a mixture of chloroform and methanol (2:1 *v*/*v*), and 16.7 μL of ammonium thiocyanate (3.94 M in water). The final mixture was vortexed, covered to prevent evaporation, and kept in the dark for 20 min for color development. Absorbance was measured at 500 nm using a Genesys 20 spectrophotometer (ThermoSpectronic, Waltham, MA, USA). Concentrations were determined from a standard curve prepared with cumene hydroperoxide.

Headspace hexanal was measured using a solid-phase microextraction gas chromatography method as described by Barden et al. [18]. A 50/30 μm divinylbenzene/carboxen/polydimethylsiloxane solid-phase microextraction fiber (Supelco, Bellefonte, PA) was inserted through the sample vial septum and exposed to the headspace for 2 min at 55 °C to absorb volatiles. The volatiles on the SPME fiber were desorbed at 250 °C for 3 min in the GC injector. The chromatographic separation was performed using a GC-2014 Shimadzu gas chromatograph equipped with a fused-silica capillary column (30 m × 0.32 mm i.d. × 1 μm) coated with 100% polydimethylsiloxane (Equity-1, Supelco, Bellefonte, PA, USA). Oven, injector, and flame ionization detector temperatures were 65, 250, and 250 °C, respectively. The split ratio was 1:7, and the run time was 10 min. Peak integration was calculated using the Shimadzu EZstart (version 7.4) software. Hexanal in methanol was added to freshly baked crackers to prepare the standard curve for the determination of hexanal concentrations.

#### 2.2.7. Characterization of Fat Mobility

1-(2,5-Dimethoxy-phenylazo)-naphthalen-2-ol (citrus red 2, CR) was used to characterize fat mobility in bulk fats and cracker dough, as explained by Du et al. [29]. Steady-state fluorescence excitation and emission spectra were recorded using a Fluoromax-4 spectrofluorometer (Horiba Scientific Inc., Edison, NJ, USA). Bulk fats were heated up to completely melt, and a 10 µL aliquot was deposited on the center of 1 mm wide quartz cuvettes. Dough samples (approximately 10 g) were placed in the bottom half of a 3 mm deep, 5 cm wide Petri plates, and a quartz slide cover was placed on top of the dough. Samples were measured in a front-face mode. The excitation wavelength was set at 520 nm. Fluorescence emission intensity in counts per second, and peak emission wavelength were determined over the range of 540 to 720 nm.

A concentration study was performed for bulk fats to determine the CR concentration that could provide a signal without exhibiting inner filter in both samples (i.e., bulk oils and dough). CCCR in bulk oils was tested at 0, 0.02, 0.04, 0.08, 0.10, and 0.15 mM, and the spectra were collected using a long-pass cut-on filter of 590 nm to reduce the contribution of scattered light. Excitation and emission slits were both 2 nm. For cracker dough, CR was tested at 0, 0.4, 0.8, and 1.2 mM, and a 550 nm long-pass filter was used to reduce scattering disturbance. Excitation and emission slits were 1 nm and 4 nm, respectively. 

The spectrum of control samples (i.e., samples without CR but same composition) was subtracted from the fluorescence emission spectra of CR-containing samples to eliminate background noise. Once the optimum CR concentration was selected, this amount was consistently used in all samples, and the emission intensity was normalized to the range of 0 to 1 by dividing the samples’ spectra by the overall maximum. 

#### 2.2.8. Confocal Microscopy

BODIPY 493/503 (1 mg) was dissolved in methanol (5 mL) and added to the fat samples (10 g). The BODIPY containing fat was then used to make crackers. Confocal images of crackers were captured using a Nikon Confocal Microscope (C1 Digital Eclipse, Tokyo, Japan) with a PL FLUOTAR ELWD 20.0 × 0.45 objective lens. BODIPY was excited at 488 nm by an air-cooled argon ion laser (Model 376 IMA1010 BOS; Melles Griot; Carlsbad, CA, USA). Emission spectra were collected at 515 ± 30 nm. The detector pinhole size was 150 μm. Confocal microscopy images were analyzed using EZ-CS1 (version 3.8) software (Nikon; Melville, NY, USA), and optical images taken on the same microscope were analyzed using NIS-Elements (version 3.0) software (Nikon, Melville, NY, USA).

#### 2.2.9. Statistical Analysis

All samples were measured in triplicate, and data represent mean values. Statistical analysis was performed using a one-way analysis of variance (ANOVA) with comparison means performed using Tukey’s honestly significant difference post hoc test (*p* = 0.05). In all cases, comparisons of the means were performed using the Minitab Express software, version 1.5.0 (State College, PA, USA). Lag phase was determined as the time between day 0 and the first data point where it and each subsequent data point was significantly greater than day 0 concentration of both lipid hydroperoxides and hexanal (*p* ≤ 0.05).

## 3. Results and Discussion 

### 3.1. Properties of Fat in Bulk Fat and Cracker Dough

In an attempt to understand how the physical characteristics of the fat in the crackers could impact oxidative stability, two blended fats were formulated to have the same linoleic acid (18:2) and tocopherol compositions but different SFCs at 55 °C, the storage temperature in this study. These were hydrogenated soybean fat + interesterified soybean fat (HI, SFC = 32.0%) and hydrogenated soybean fat + sunflower oil (HS, SFC = 24.9%; Table 1). Since blending could not make all fatty acid concentrations the same, we focused on linoleic acid (18:2), which is the polyunsaturated fatty acid at the greatest concentration (20%) and is much more susceptible to oxidation than oleic acid (18:1). Tocopherols were added to attain the same concentrations of each tocopherol homolog (488.5 ppm total tocopherols, Table 1) in all fat blends. 

SFC is typically determined in bulk oils. However, food components can impact fat crystallization (e.g., free fatty acids, phospholipids, and sterols) [30]. Hence, the amount of solid fat in bulk oils could be different than that in foods such as crackers. To test this, we characterized the micro-viscosity of the fat phase with a lipophilic fluorescent molecular rotor. 1-(2,5-Dimethoxy-phenylazo)-naphthalen-2-ol (citrus red 2, CR) has been successfully used to characterize microenvironments in pure triglycerides and their mixtures with different SFC [29]. CR is a molecular rotor, which consists of two segments that, upon photoexcitation, can rotate relative to the other depending on the rigidity of the surrounding environment. In a low-viscosity environment, the excited CR is predominately in a twisted intra-molecular charge transfer (TICT) state, from which it decays non-radiatively. In a high-viscosity or rigid environment, steric hindrance deters the formation of a TICT state, and CR exhibits a locally excited (LE) intramolecular charge-transfer state (ICT) that favors radiative decay to the ground state and fluorescence emission.

Initially, the ability of the probe to determine the micro-viscosity of fat blends was tested using the hydrogenated soybean + sunflower oil blend (HS). CR was excited at 520 nm, and its maximum emission peak was identified at 600 nm. The emission intensity of CR increased linearly with increasing CR concentrations of 0 to 0.1 mM (data not shown). At higher CR concentrations (>0.1 mM), the emission intensity plateaued, which is typical of an inner filter effect [31,32], i.e., re-absorbance of the emitted fluorescence. Therefore, 0.1 mM CR was chosen to study micro-viscosity in all the blended fats. 

**Figure 1:**
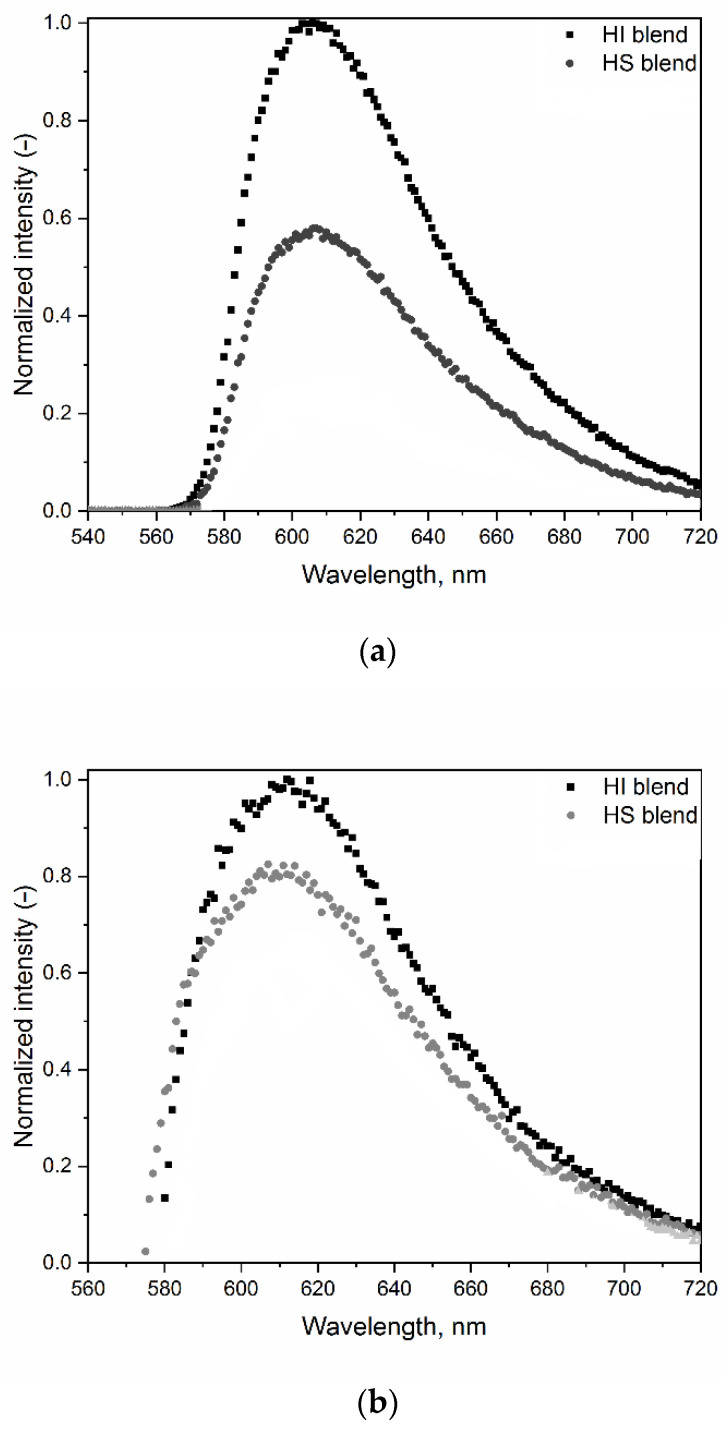
(**a**) Normalized fluorescence emission spectra of 0.1 mM citrus red 2 (CR) in hydrogenated soybean fat + interesterified soybean fat blend (HI, 52.3% solid fat content at room temperature) and hydrogenated soybean fat + sunflower oil blend (HS, 43.8% solid fat content at room temperature) (**b**) Normalized fluorescence emission spectra of 1.2 mM citrus red 2 (CR) in the lipid phase of cracker doughs made from these fats.

Fats with higher solid fat content restricted the movement of entrapped liquid fat such that the radiative decay of CR was favored, and higher emission intensity was observed (Figure 1a). Emission intensity of CR in bulk fats was greater in HI (52.3% SFC at room temperature) than in HS (43.8% SFC at room temperature) blends. As mentioned, CR is a fat-soluble probe successfully used to characterize microenvironments in pure triglycerides [29]. In this study, CR emission intensity increased with increasing SFC, indicating that CR could also determine the micro-viscosity of commercially available fats. 

In baked crackers (with and without CR), background fluorescence in the range needed to study the probe (540 to 720 nm) was too high, so CR emission in baked samples could not be used to determine the properties of the fat (data not shown). This could be due to Maillard products that fluoresce in the same wavelength range [33,34]. Therefore, CR emission was measured in the cracker dough prior to cooking. Because CR emission is concentration dependent and could be matrix dependent, different CR concentrations were again examined to determine an appropriate concentration to study in cracker dough. CR emission in the cracker dough increased linearly up to a concentration of 1.2 mM (data not shown), so this concentration was used in the cracker doughs made from the different fat blends. CR emission intensity in the cracker dough was again greater for HI (52.3% SFC) than HS (43.8% SFC) (Figure 1b). 

Compared to CR emission intensity in bulk fat (maximum intensity around 600 nm), the emission intensity peak of CR in cracker dough was shifted toward a longer wavelength (maximum intensity around 610 nm). A red shift in the CR emission peak was reported as environmental polarity increased [28]. In cracker matrices, lipid/carbohydrate and lipid/protein interfaces were observed using confocal microscopy [18]. These interfaces would likely have a higher polarity than lipids measured alone. The CR’s red shift in cracker dough could be attributed to the CR located at these interfaces.

In bulk fats, the HI blend (52.3% SFC) had a normalized maximum intensity two times higher than the HS blend (43.8% SFC), whereas there was only a 20% difference in the cracker dough. The normalized intensity difference indicated that from the same fat, the micro-environment of the fat phase in cracker dough was not similar to the micro-environment in the bulk fat. 

In short, the fluorescence technique using CR as a probe successfully characterized the micro-viscosity of fat in both bulk fats and fat in cracker doughs. CR emission intensity increased with the increasing SFC of fat blends, tested in both bulk fats and fat phase in cracker doughs.

### 3.2. Oxidative Stability of Crackers Made from Fat Blends with Varied Solid Fat Contents 

Lipid oxidation in foods is impacted by many different factors, including fatty acid composition, prooxidant type and concentration (e.g., metals and oxygen), and antioxidant type and concentration (e.g., tocopherols). In addition, the physical state of a fat can impact its oxidative stability. For example, SFC could impact oxidization if it results in separation of fat phases, with one phase being more highly unsaturated than the total fat and thus more susceptible to oxidation.

**Figure 2:**
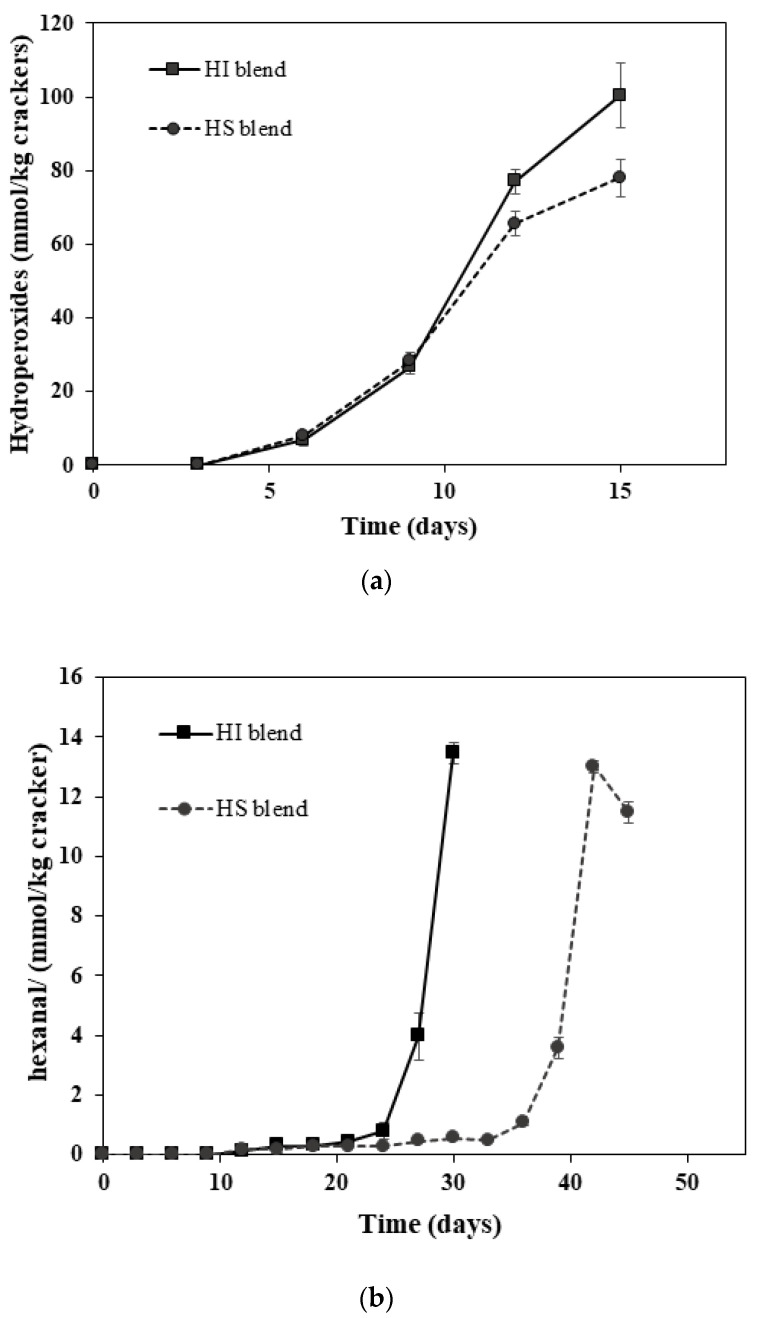
Formation of (**a**) lipid hydroperoxides and (**b**) headspace hexanal in crackers made with hydrogenated soybean fat + interesterified soybean fat (HI, SFC = 32.0%) or hydrogenated soybean fat + sunflower oil (HS, SFC = 24.9%). Data points represent means (*n* = 3) ± standard deviations. Some standard deviations lie within the data points.

To determine how solid fat content impacted lipid oxidation, the oxidative stability of crackers made with fat blends with different SFCs but the same linoleic acid and tocopherol concentrations was evaluated. We assumed that both samples would have similar prooxidant concentrations, as all had the same level of non-fat ingredients that contained prooxidants such as iron and were exposed to the same level of oxygen during processing and storage. The HI blend had a SFC of 32.0%, while the HS blend had a SFC of 24.9% at the 55 °C incubation temperature. Crackers made from the two fat blends had the same hydroperoxides lag phase (9 days) but different hexanal lag phases (Figure 2). The HI blend, which had a higher SFC, resulted in a shorter hexanal lag phase (24 days) than the HS blend, which had a lag phase of 33 days. The fact that hexanal formation but not hydroperoxides formation was different suggests that hydroperoxide decomposition into hexanal was being impacted. HI had slightly higher levels of linolenic acid, but this would not impact our hexanal observations, as linolenic acid does not decompose into hexanal. This could be due to the ability of the hydroperoxides to interact with prooxidants such as transition metals in the non-lipid fraction. The crackers with the high solid fat content HI could have a lipid phase that was more concentrated in unsaturated fatty acids. This could allow for higher oxidation rates and concentration of hydroperoxides, which could then accumulate at the lipid interface, where they could be decomposed into hexanal. Similar trends have been observed in oil-in-water emulsions, where the presence of solid fat pushes unsaturated fats to the droplet surface, where they oxidize faster [10,11,12,35].

**Figure 3:**
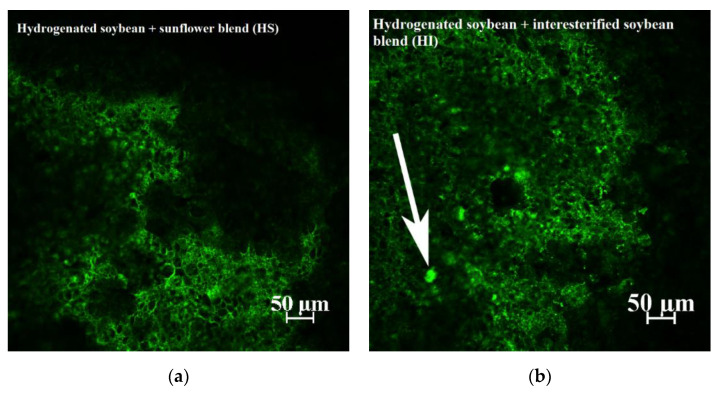
Confocal images of crackers made with either (**a**) hydrogenated soybean fat + interesterified soybean fat (HI, SFC = 32.0%) or (**b**) hydrogenated soybean fat + sunflower oil (HS, SFC = 24.9%).

As was observed by Barden et al. [18], the fat (green) formed a continuous phase surrounding the starch granules (black) in crackers with either the HI or HS blends (Figure 3). The HI fat seemed to have more high-density fat areas (indicated by the arrow). While not definitive, the crackers made with HI could have more fat crystals high in saturated fatty acids, which could produce a separate, higher unsaturated fat phase that was more susceptible to oxidation. 

### 3.3. Oxidative Stability of Crackers Made from a Fat Blend or Interesterified Soybean Fat 

During the interesterification process, antioxidants may be removed, and loss of endogenous antioxidants, such as tocopherols, can explain the lower oxidative stability of interesterified fats [7,36]. In an attempt to better understand how oxidation differed in crackers made with blended fats (fully hydrogenated soybean oil and soybean oil) versus interesterified soybean oil, crackers were made with a fat phase that was formulated to have similar linoleic acid concentrations: 43.30% for the fat blend and 43.27% for the interesterified fat (Table 1). Again, tocopherols were added to the fats to make the concentrations of each tocopherol homolog the same (1073.8 ppm total tocopherols, Table 1). On the other hand, SFC was difficult to equalize, but they were similar at 17.4 and 13.7% at 55 °C for fat blend and interesterified fat, respectively.

**Figure 4:**
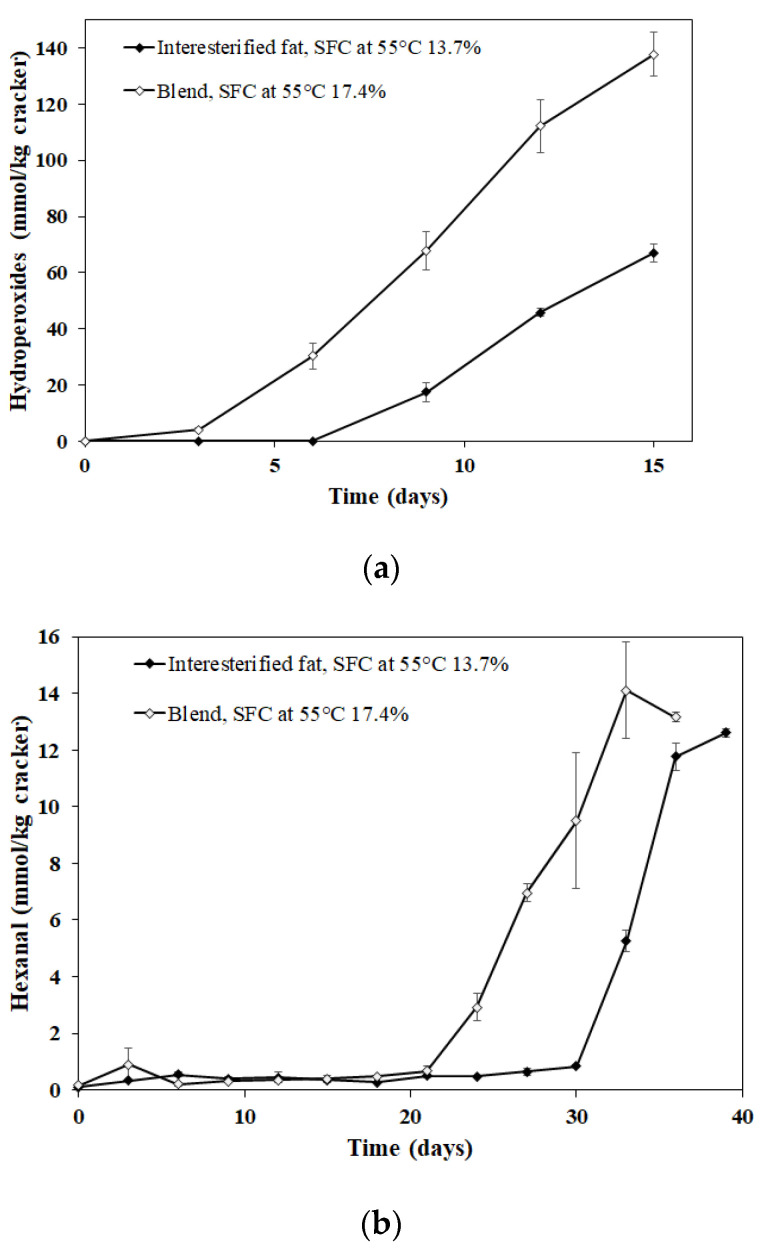
Formation of (**a**) lipid hydroperoxides and (**b**) headspace hexanal in crackers made from fat blend (17.4% solid fat content) and interesterified fat (13.7% solid fat content), which had similar fatty acid profile and tocopherol profile at 55 °C. Data points represent means (*n* = 3) ± standard deviations. Some standard deviations lie within the data points.

The crackers made with the interesterified fat were more oxidatively stable than those made with the fat blend, as determined by both lipid hydroperoxides and headspace hexanal (Figure 4). The lag phase of the interesterified fat was 3 and 9 days longer than the crackers with the fat blend for lipid hydroperoxides and headspace hexanal, respectively. This could be due to the liquid oil separating from the solid hydrogenated fat in the fat blend, thus producing a concentrated source of oxidizable unsaturated fatty acids. Conversely, the unsaturated fatty acids in the interesterified fat would be distributed more evenly throughout the triacylglycerols, so there would not be as discrete differences in liquid and solid fat fractions as in the fat blend. In a way, the interesterified fat dilutes the unsaturated fatty acids throughout the fat, making them less susceptible to oxidation, as is seen in liquid oil blends containing saturated (e.g., medium chain triglycerides) and unsaturated fatty acids (e.g., fish oil) [6]. The hydroperoxide and hexanal lag phase for the fully hydrogenated soybean oil and soybean oil in this experiment (3 and 21 days, respectively) was shorter than in previous experiments with fat blends, which was likely due to the higher linoleic acid concentrations used in this experiment (43 vs. 20%). In addition, the crackers with the interesterified fat had a lag phase similar to the HS fat blend (32.8% fully hydrogenated soybean oil and 67.2% sunflower oil) even though it had over twice the amount of linoleic oil (43% vs. 20%), suggesting that interesterified fats could be used to improve the nutritional profile of low-moisture foods by allowing food manufacturers to increase the level of healthy unsaturated fatty acids.

**Figure 5:**
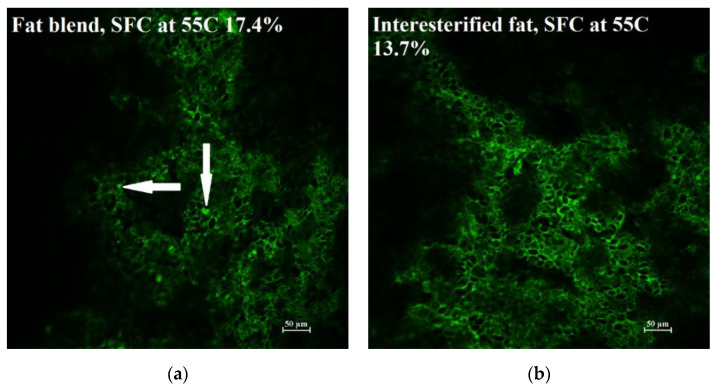
Confocal images of crackers made with either (**a**) a fully hydrogenated soybean fat (17.2%) and soybean oil (82.8%) blend or an (**b**) interesterified soybean oil.

Confocal microscopy was also used to evaluate if differences existed in the physical structure of the crackers made from a fat blend and interesterified fat (Figure 5). The crackers made with the fat blend had small areas of high-density fat (indicated by arrows), as seen in the HI fat blend (Figure 3). However, in this case, these high-density fat structures were less obvious, likely due to the lower amount of fully hydrogenated soybean oil (17.2%) used in this experiment compared to the HI experiment, which had 53.8% fully hydrogenated soybean oil. These areas of high fat density are likely solid fat, suggesting that the remaining fat in the cracker would be more unsaturated, yielding to faster oxidation rates. 

## 4. Conclusions

Using a cracker system made with fats of varying solid fat compositions but similar polyunsaturated fatty acid and tocopherol composition, it was possible to determine how the physical properties of lipids impact lipid oxidation in a low-moisture matrix. Overall, higher SFC increased oxidation rates even when polyunsaturated fatty acids content was the same. The most likely reason for this observation is that the greater percentage of solid fat created a lipid phase that was more concentrated in polyunsaturated fat and thus became more susceptible to oxidation. It was also observed that crackers made with interesterified fats were more oxidatively stable than a fat blend at the same linoleic acid and tocopherol concentration. This could be due to the polyunsaturated fatty acids in the interesterified fat being more evenly distributed throughout all triacylglycerols compared to the liquid oil in the fat blend, which would have more discrete liquid oil fraction. This suggests that interesterified fats could be used to decrease lipid oxidation rates more effectively than fat blends and thus increase shelf life. In addition, this could help improve the nutritional profile of low-moisture foods, as higher levels of unsaturated fatty acids could be incorporated into a product with an interesterified fat with less of an impact on oxidative rancidity.

## Figures and Tables

**Table 1:** Solid fat content (SFC), fatty acid profile, and tocopherol profile of a series of two fat blends and a comparison of fat blends and interesterified fat that were used to make the crackers.

Fat		53.8% Fully Hydrogenated Soybean Fat + 46.2% Interesterified Soybean Fat (HI)	32.8% Fully Hydrogenated Soybean Fat + 67.2% Sunflower Oil (HS)	17.2% Fully Hydrogenated Soybean Fat + 82.8% Regular Soybean Oil	Interesterified Soybean Fat
SFC at 55 °C, %		32.00	24.90	17.40	13.70
SFC at 25 °C, %		52.30	43.80	36.60	32.00
Fatty acid profile, %	14:0	0.0	0.00	0.17	0.00
	16:0	11.61	7.00	11.50	11.06
	16:1	0.04	0.08	0.12	0.08
	18:0	56.46	30.32	18.18	22.19
	18:1	6.86	40.41	17.69	14.84
	18:2	20.00	20.00	43.30	43.27
	18:3	3.01	0.18	5.82	6.52
Alpha-tocopherol, ppm		200.78	200.78	53.72	53.72
Beta-tocopherol, ppm		0.00	0.00	0.00	0.00
Gamma-tocopherol, ppm		254.53	254.53	862.86	862.86
Delta-tocopherol, ppm		33.10	33.10	157.23	157.23

## Data Availability

Data are contained within the article.

## References

[1] Atkinson G. (2011). 12-Fats and oils as biscuit ingredients A2-Manley, Duncan. Manley’s Technology of Biscuits, Crackers and Cookies.

[2] McClements D.J., Decker E.A., Damodaran S., Parkin K.L., Fennema O.R. (2008). Lipids. Fennema’s Food Chemistry.

[3] Bhatnagar A.S., Prasanth Kumar P.K., Hemavathy J., Gopala Krishna A.G. (2009). Fatty Acid Composition, Oxidative Stability, and Radical Scavenging Activity of Vegetable Oil Blends with Coconut Oil. J. Am. Oil Chem. Soc..

[4] Frankel E.N., Huang S.W. (1994). Improving the oxidative stability of polyunsaturated vegetable oils by blending with high-oleic sunflower oil. J. Am. Oil Chem. Soc..

[5] Wang J., Shahidi F. (2017). Oxidative stability of marine oils as affected by added wheat germ oil. Int. J. Food Prop..

[6] Culler M.D., Inchingolo R., McClements D.J., Decker E.A. (2021). Impact of Polyunsaturated Fatty Acid Dilution and Antioxidant Addition on Lipid Oxidation Kinetics in Oil/Water Emulsions. J. Agric. Food Chem..

[7] Martin D., Reglero G., Señoráns F.J. (2010). Oxidative stability of structured lipids. Eur. Food Res. Technol..

[8] Neff W.E., El-Agaimy M.A., Mounts T.L. (1994). Oxidative stability of blends and interesterified blends of soybean oil and palm olein. J. Am. Oil Chem. Soc..

[9] Neff W.E., Selke E., Mounts T.L., Rinsch W., Frankel E.N., Zeitoun M.A.M. (1992). Effect of triacylglycerol composition and structures on oxidative stability of oils from selected soybean germplasm. J. Am. Oil Chem. Soc..

[10] Okuda S., McClements D.J., Decker E.A. (2005). Impact of lipid physical state on the oxidation of methyl linolenate in oil-in-water emulsions. J. Agric. Food Chem..

[11] Yucel U., Elias R.J., Coupland J.N. (2013). Effect of Liquid Oil on the Distribution and Reactivity of a Hydrophobic Solute in Solid Lipid Nanoparticles. J. Am. Oil Chem. Soc..

[12] Yucel U., Elias R.J., Coupland J.N. (2013). Localization and reactivity of a hydrophobic solute in lecithin and caseinate stabilized solid lipid nanoparticles and nanoemulsions. J. Colloid Interface Sci..

[13] Ali H., El-Sayed K., Sylvester P.W., Nazzal S. (2010). Molecular interaction and localization of tocotrienol-rich fraction (TRF) within the matrices of lipid nanoparticles: Evidence studies by Differential Scanning Calorimetry (DSC) and Proton Nuclear Magnetic Resonance spectroscopy ((1)H NMR). Colloids Surf. B Biointerfaces.

[14] Helgason T., Awad T.S., Kristbergsson K., Decker E.A., McClements D.J., Weiss J. (2009). Impact of surfactant properties on oxidative stability of beta-carotene encapsulated within solid lipid nanoparticles. J. Agric. Food Chem..

[15] McClements D.J., Decker E.A., Weiss J. (2007). Emulsion-based delivery systems for lipophilic bioactive components. J. Food Sci..

[16] Salminen H., Helgason T., Kristinsson B., Kristbergsson K., Weiss J. (2013). Formation of solid shell nanoparticles with liquid omega-3 fatty acid core. Food Chem..

[17] Labuza T.P., Tannenbaum S.R., Karel M. (1970). Water content and stability of low-moisture and intermediate-moisture foods. Food Technol..

[18] Barden L., Vollmer D., Johnson D., Decker E. (2015). Impact of iron, chelators, and free fatty acids on lipid oxidation in low-moisture crackers. J. Agric. Food Chem..

[19] Vu T.P., He L., McClements D.J., Decker E.A. (2020). Effects of water activity, sugars, and proteins on lipid oxidative stability of low moisture model crackers. Food Res. Int..

[20] Laguerre M., Lopez Giraldo L.J., Lecomte J., Figueroa-Espinoza M.C., Barea B., Weiss J., Decker E.A., Villeneuve P. (2010). Relationship between hydrophobicity and antioxidant ability of “phenolipids” in emulsion: A parabolic effect of the chain length of rosmarinate esters. J. Agric. Food Chem..

[21] Frankel E.N., Huang S.-W., Kanner J., German J.B. (1994). Interfacial Phenomena in the Evaluation of Antioxidants: Bulk Oils vs. Emulsions. J. Agric. Food Chem..

[22] Gumus C.E., Decker E.A. (2021). Oxidation in Low Moisture Foods as a Function of Surface Lipids and Fat Content. Foods.

[23] Manzocco L., Romano G., Calligaris S., Nicoli M.C. (2020). Modeling the Effect of the Oxidation Status of the Ingredient Oil on Stability and Shelf Life of Low-Moisture Bakery Products: The Case Study of Crackers. Foods.

[24] FDA (2018). Substances Generally Recognized as Safe.

[25] Shantha N.C., Napolitano G.E. (1992). Gas-chromatography of fatty-acids. J. Chromatogr. A.

[26] Wang W., Yang J., Qi W., Yang H., Wang C., Tan B., Hammock B.D., Park Y., Kim D., Zhang G. (2017). Lipidomic profiling of high-fat diet-induced obesity in mice: Importance of cytochrome P450-derived fatty acid epoxides. Obesity.

[27] Márquez A.L., Pérez M.P., Wagner J.R. (2013). Solid Fat Content Estimation by Differential Scanning Calorimetry: Prior Treatment and Proposed Correction. J. Am. Oil Chem. Soc..

[28] Shantha N.C., Decker E.A. (1994). Rapid, sensitive, iron-based spectrophotometric methods for determination of peroxide values of food lipids. J. AOAC Int..

[29] Du H., Kim C., Corradini M.G., Ludescher R.D., Rogers M.A. (2014). Micro-viscosity of liquid oil confined in colloidal fat crystal networks. Soft Matter.

[30] Ribeiro A.P.B., Masuchi M.H., Miyasaki E.K., Domingues M.A.F., Stroppa V.L.Z., de Oliveira G.M., Kieckbusch T.G. (2015). Crystallization modifiers in lipid systems. J. Food Sci. Technol..

[31] Alhassawi F.M., Corradini M.G., Rogers M.A., Ludescher R.D. (2018). Potential applications of luminescent molecular rotors in food science and engineering. Crit. Rev. Food Sci. Nutr..

[32] Jameson D.M. (2014). Introduction to Fluorescence.

[33] Birlouez-Aragon I., Nicolas M., Metais A., Marchond N., Grenier J., Calvo D. (1998). A Rapid Fluorimetric Method to Estimate the Heat Treatment of Liquid Milk. Int. Dairy J..

[34] Leclère J., Birlouez-Aragon I. (2001). The Fluorescence of Advanced Maillard Products Is a Good Indicator of Lysine Damage during the Maillard Reaction. J. Agric. Food Chem..

[35] Tikekar R.V., Nitin N. (2011). Effect of physical state (solid vs. liquid) of lipid core on the rate of transport of oxygen and free radicals in solid lipid nanoparticles and emulsion. Soft Matter.

[36] Kowalska D., Kostecka M., Tarnowska K., Kowalski B. (2014). Oxidative stabilities of enzymatically interesterified goose fat and rapeseed oil blend by rancimat and PDSC. J. Therm. Anal. Calorim..

